# The impact of amblyopia, reduced viewing conditions and binocular vision on reading ability: a narrative review

**DOI:** 10.3389/fnins.2026.1838192

**Published:** 2026-06-26

**Authors:** Nicole A. Dranitsaris, Alexandre Reynaud

**Affiliations:** 1 McGill Vision Research, Department of Ophthalmology and Visual Sciences, McGill University, Montreal, QC, Canada; 2 Centre for Digital Brain Therapies, BRaIN Program, Research Institute of the McGill University Health Centre, Montreal, QC, Canada

**Keywords:** amblyopia, binocular vision, low vision, reading, review

## Abstract

Amblyopia is a neurodevelopmental disorder of the visual cortex which leads to issues in visual acuity, contrast sensitivity and eye movement patterns, all characteristics which, along with other deficits may negatively influence reading skills. Individuals with reduced reading capabilities due to amblyopia or other binocular vision disorders may have difficulties navigating various social aspects of daily life including employment and academics. Reading is an active viewing task which involves multiple oculomotor and cognitive processes. In this review, we introduce how a lack of binocular vision or other low vision issues such as blur, low illumination or altered contrast sensitivity may contribute to impaired reading performance. These impairments, particularly in amblyopia, include changes to reading speed, eye movements and crowding. Though reading is such an important daily skill, and is known to be impacted in amblyopia, there are no known treatments for this condition which are specifically designed to improve reading. Binocular therapies may be leveraged to address these issues. This narrative review provides available evidence on alterations to reading ability in amblyopia and why this may be relevant for developing novel amblyopia therapies.

## 1 Introduction

Affecting between 1 and 5% of the global population (Pescosolido et al., 2014; Fu et al., 2020), amblyopia, or lazy eye, is a binocular vision disorder of the visual cortex (Hess and Thompson, 2015). During development, altered vision in one eye such as anisometropia, strabismus, or more rarely, monocular deprivation (Von Noorden, 1974; Levi, 2021) lead to incompatible inputs from the two eyes when the visual signals reach the visual cortex, where binocular combination should occur. To mitigate the impact of the deteriorated vision from the bad eye, the amblyopic brain *suppresses* the input from the bad eye in favour of the input from the good eye. This causes the binocular circuits in the brain to develop abnormally, resulting in a wide range of irregularities: poor visual acuity, stereovision, contrast sensitivity, altered eye movement patterns, crowding and even changes to fellow eye function, though there is a wide range of symptoms and severities amongst individuals (Levi, 2021; Jefferis et al., 2015; McKee et al., 2003; Kelly et al., 2023). All these issues may affect reading ability, an everyday task, critical for development. Studies have found that children and adults with amblyopia read more slowly, have greater fixation instability and have altered eye movement patterns during reading compared to controls (Bhutada et al., 2022; Stifter et al., 2005) which was associated with their worse visual acuity and stereoacuity. Another study also found that children with amblyopia believed they were less scholastically competent which was correlated with them having slower reading speed (Birch et al., 2019).

Thus, we conducted a narrative literature review, of which the aim was to address how factors such as binocular vision, low vision characteristics and more specifically amblyopia impacts reading skills. No systematic methodology was employed. We will first present the functional and social barriers associated with reduced reading ability. Then we will introduce the oculomotor mechanisms underlying normal reading and discuss the advantages of reading with two eyes compared to one eye. There will be subsequent details on the impact of low vision characteristics, such as blur, contrast and luminance on reading ability. This will lead into the discussion of specifically how amblyopia impacts reading speed, eye movements and crowding during reading. Finally, we will examine how, in that light, reading could be used as a framework for therapies.

## 2 Social challenges associated with reading for people with amblyopia

The task of reading is integral for learning, creative thinking, and is involved in virtually every aspect of daily life (Birch and Kelly, 2017). It is an important aspect of human development which helps people learn about themselves, society and even interpretation of social cues (Freire, 1983).

Reduced reading capabilities will impact the ability of individuals to advance their life in the modern world (Birch and Kelly, 2017). They may be impacted academically, encounter difficulties securing employment, and even face challenges when navigating the world. One study reported that amblyopic children themselves believe they have lower scholastic and social skills than control children. Worse scholastic competency was related to slower reading speeds (Birch et al., 2019). Such lack of self-competency may impact how they then are able to thrive in academic and social situations. The importance of literacy for daily life is highlighted by the fact that various education governing bodies require timed standardized tests to ensure that generations of students have basic reading and writing skills. For example, the province of Ontario, Canada requires that all students pass the Ontario Secondary School Literacy Test to graduate high school (Education Quality and Accountability Office, n.d.). However, such tests and modern life in general do not consider the difficulties that people with vision impairments might face when completing them. While in provinces of Canada, such as Ontario, it is possible for a child to receive accommodation even without a formal disability diagnosis (Government of Ontario, n.d.), studies report that in other countries such as the United States, students require a disability diagnosis of visual impairment to receive accommodations. As a visual impairment is defined as having at most 20/40 visual acuity in the strong eye, many people with amblyopia would not qualify (Birch and Kelly, 2017). So, for those with reduced visual capacities, reading, and other tasks in daily life become more difficult to navigate (Candy and Cormack, 2022). People with binocular vision problems, such as amblyopia or convergence insufficiency, are often treated for specific visual impairments such as poor visual acuity or poor stereoacuity, but there is a lack of therapies that aim to improve reading ability specifically, even though there are known deficits in these conditions.

## 3 The process of reading

Reading is a dynamic skill which involves a combination of various perceptual, motor, and cognitive processes across multiple brain regions including frontal, parietal, occipital and temporal lobes. Reading, a relatively recent human activity compared to spoken language, is not an innate skill but rather acquired through learning. Learning to read has not only been observed to reshape brain structure and connectivity during development (Xu et al., 2020; Dehaene et al., 2010; Houston et al., 2014; Justino and Kolinsky, 2023) but is needed for creativity, education, and many other modern skills (Birch and Kelly, 2017). If reading ability is impaired it will become significantly more difficult for an individual to succeed in almost every aspect of daily life (Houston et al., 2014; Birch and Kelly, 2017). Amblyopes, who also have impaired binocular vision, may face these educational and even directional issues, as modern life in general is designed for people with binocular vision (Candy and Cormack, 2022). The primary processes that occur during binocular reading are (1) arranging and performing proper oculomotor patterns so text may be inputted into the brain and, (2) deriving the meaning of the text being read (Radach et al., 2004).

### 3.1 Oculomotor processes in reading

In general, the first step in the process of successful reading involves a fixation period, where words can be focused on the fovea so they can then be processed and understood (Rayner, 1995; Rayner, 1986). These fixational periods last on average 200–250 milliseconds (Rayner, 1995). Most visual information, particularly that which is fine or detailed, such as text, is processed by the fovea, which subtends approximately 2° of visual angle, or approximately 8 letters from the fixation point in conventional reading conditions (Rayner, 1986), and may generalize across a reasonably wide range of print sizes (Legge and Bigelow, 2011). The next step is the movement made to the next fixation point, called a saccade. No beneficial visual information is retained during a saccade due to the high speed at which they occur (Taylor, 1965; Wolverton and Zola, 1983). Approximately 10–15% of saccades are *regressive*, meaning a reader must move their eyes back to the previous text as it was not properly input (Rayner, 1995).

Eye movements patterns may also depend on the cognitive processes occurring during reading, i.e., whether words are orthographically processed as a whole or if they are phonologically processed by syllables (Caylak, 2010; Coltheart et al., 2001; Grainger, 2018; Gabrieli et al., 2010; Radach et al., 2004; Siegel, 1993; White, 2008; Wong and Chen, 1999). To our knowledge there is no evidence about how these processes may be distinctively impacted in amblyopia.

## 4 Binocular advantages while reading

As with other daily viewing tasks, many studies have found that reading binocularly provides the advantage of efficacy by aiding the individual to perceive one stable input of the text. The equal use of both eyes allows for improved processing of visual input that is required to read efficiently (Nikolova et al., 2017; Jainta and Joss, 2019). A study by Johansson et al. (2014), where participants read standardized paragraphs of text from the International Reading Speed Texts (IReST) monocularly in each eye and binocularly demonstrated that at various text contrasts, individuals had fixation periods that were 8.9% increased in duration monocularly (averaged between both eyes) compared to binocularly. They also read slower monocularly with the non-dominant eye (measured at distance) than binocularly (9 less words per minute monocularly), effects which increased as contrast decreased. Jainta et al. (2014), showed a similar result on an experiment where they had individuals read short sentences of red text on a black background in randomized order. Here, participants read sentences fully binocularly, monocularly, or using saccade-contingent viewing changing, partially binocularly or monocularly and then switching to the other viewing condition. Monocular left and right eye viewing were counterbalanced. They found shorter mean binocular sentence reading times (6.3% faster) and, using eye tracking, shorter binocular fixation periods (6.5% faster) compared to monocular reading. They also demonstrated that lexical identification of frequently viewed words on the first fixation period can only occur with binocular viewing and that monocular only viewing provided no advantage when viewing high frequency words compared to low frequency ones and had overall less efficient lexical processing. This is significant in demonstrating how cognition and binocular vision, or lack thereof, can significantly impact eye movement patterns while reading.

These studies, along with various others investigating binocular advantages for reading, (Jainta and Jaschinski, 2012; Sheedy et al., 1986; Heller and Radach, 1999) show quicker reading times, less fixations, and less regressive saccades compared to monocular reading (Nikolova et al., 2017). It is worth noting that though it may be thought that they eyes fixate on the same point, more recent research has found, even in normal binocular vision, that the left eye tends to fixate slightly above and more to the right than the right eye (Nuthmann and Kliegl, 2009). In general, though, these binocular vision studies suggest that at least the binocular advantage is linked to low level visual processing where binocular fusion takes place and the independent inputs from each eye are combined. This will give the reader more relevant visual information from which they can detect and comprehend the text (Jainta and Joss, 2019). In other words, binocular reading improves the quality of visual representations a reader can use to derive meaning from text (Jainta et al., 2014).

The binocular advantage of reading is especially evident in studies with individuals affected by visual problems in one or both eyes that were simulated artificially (Yu et al., 2022; Yu and Kwon, 2023) or originated naturally (Kanonidou et al., 2010; Kelly et al., 2015; Stifter et al., 2005; Bhutada et al., 2022; Jainta and Joss, 2019; Benhaim-Sitbon et al., 2023). This demonstrates the importance of developing new tools for people with low vision that may improve their binocular vision and give them greater reading ability.

## 5 Reading ability is impacted under reduced viewing conditions

In daily life, there are many instances where altered viewing conditions may be encountered. Such conditions caused by the natural environment, visual or neurological disorders may make it increasingly difficult for individuals to read effectively (Ram and Bhardwaj, 2018; Yu et al., 2022; Benhaim-Sitbon et al., 2023). Even for those with normal vision, reading when there is poor lighting, when text is at low contrast or blurry, becomes more challenging (Yu et al., 2022). How these factors affect daily tasks, like reading, should all be taken into consideration when developing treatments for people who have low vision.

### 5.1 Blur

**Figure 1:**
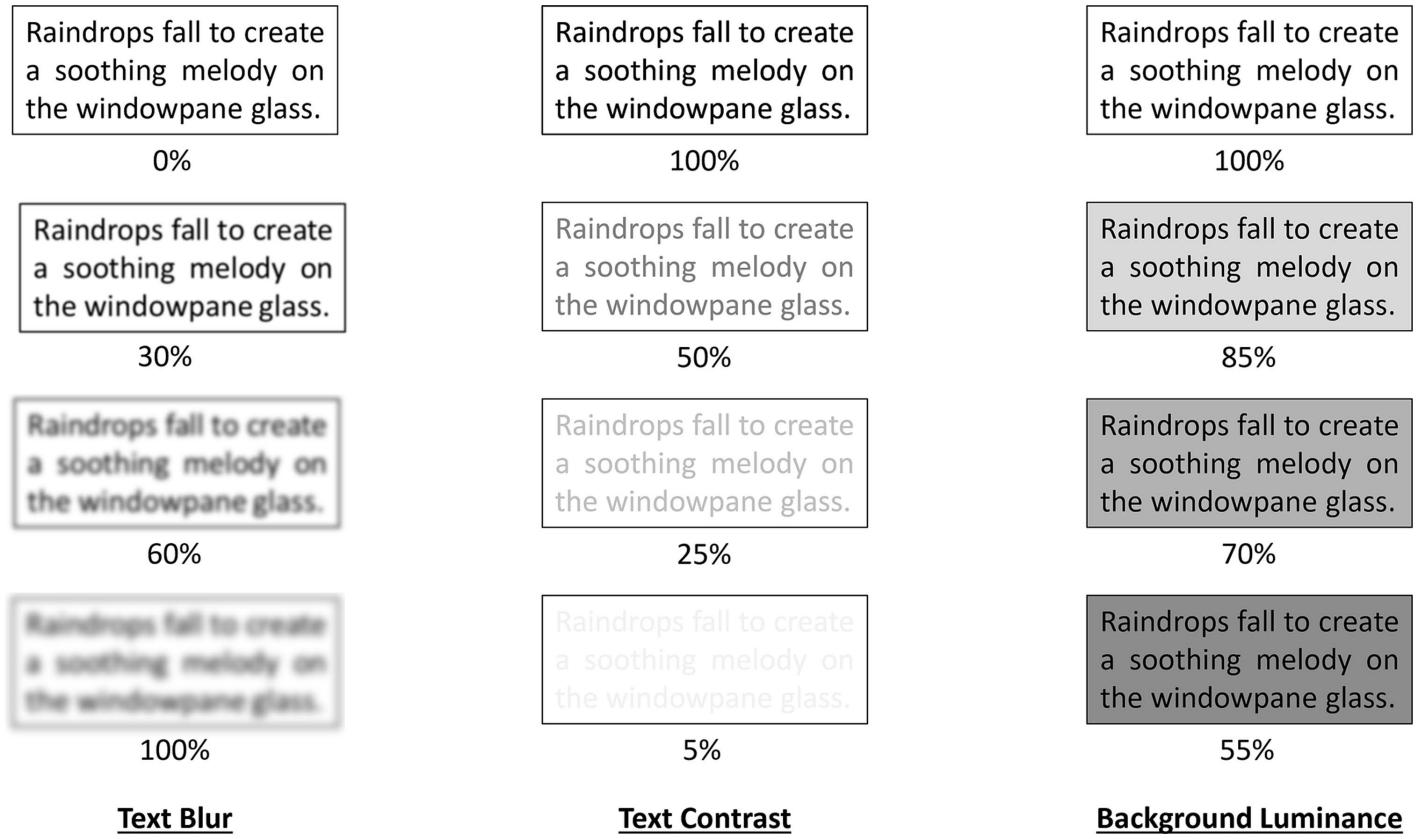
Example of various altered viewing conditions that may impact reading ability including text blur (arbitrary unit), contrast, and background luminance at different levels with 100% referring to highest level.

Blur is a known factor to affect reading performance. Examples of various blur levels can be found in the left-most column of Figure 1. Legge et al. (1985) discovered that lower blur levels, allowed observers to have faster reading speeds up to 2 cycles/letter. Chung et al. (2007) found that, in normal sighted participants, reading ability was significantly affected only at the highest level of dioptric blur used, 3 diopters. Their maximum reading speed, the fastest someone can read regardless of print size, on the MNREAD test (Mansfield and Legge, 2007), was reduced from 177.6 words per minute (wpm) at no blur to 136.2 wpm. Participants also had increased threshold print sizes worse, reading acuity and visual acuity at the highest blur level. Interestingly, these factors were not affected during reading at lower blur levels, meaning that people are able to process blurred text, unless it is extremely severe (Chung et al., 2007). In accordance with this, Yu et al. (2022) recently demonstrated that in individuals with normal vision, when increasing text blur, reading speed and eye movements are altered. They showed that most severely blurred text caused a reduction in reading speed by 59%. It also decreased saccade amplitude and velocity indicating that when text is blurred saccade patterns become slower and shorter. Blurry text increased the proportion of regressive saccades by 91% and the number and duration of fixation periods by 42% (Yu et al., 2022). Overall, these studies generally conclude that blur can negatively impact reading skills, but in most cases, only at the highest levels.

### 5.2 Contrast

When reading text with reduced contrast, it also becomes more difficult for individuals to read effectively. An early study by Legge et al. (1987), demonstrated that normal vision is flexible to contrast changes between 1.0 and 0.1 log units. Below this, reading speed decreases quickly. This may be illustrated in the middle column of Figure 1, when comparing 100% contrast to 50% or even 25%, the text is still generally readable to the unaltered eye. However, the 5% contrast text may become significantly more difficult to read for those with unaffected vision. Contrast also becomes an important factor when letter size reaches extremes in either direction. Thus, contrast may play a more significant role, at least for some low vision observers. As shown by Rubin and Legge (1989), the contrast sensitivity of participants was found to correlate with the effect of contrast on reading ability. Contrast of visual input was even found to affect the processing of text in a study investigating event-related brain potentials (ERPs). Participants were presented high and low contrast text and were asked to make judgements about errors in the sentences. While contrast had little effect on lower visual processes, semantic processing was found to be delayed and take longer in the low contrast condition, as the typical N400 centroparietal negativity spike was found to start approximately 100 ms later and last longer than normal (Gunter et al., 1999). In a more recent study where participants were reading standardized sentences of text in 100% and reduced contrasts aloud, reading speed, saccade amplitude and velocity were found to decrease between the highest and lowest contrast. Moreover, fixation duration as well as the number of fixations increased. The proportion of regressive saccades also became significantly higher when contrast was reduced beyond 2.66%, increasing from 25% to over 30% (Yu et al., 2022). Generally, it can be concluded that across studies vision appears to be functional across a wide range of contrasts, but reading is affected at the extreme.

### 5.3 Luminance

In the photopic range, background and environmental luminance changes have little effect on reading ability. Examples of different background screen illuminations can be found in the right-most column of Figure 1. The normal eye can function effectively at various photopic luminance levels in the environment (Kalloniatis and Luu, 2005). One study by Yu et al. (2022), found no significant changes in reading speed, saccade velocity, number of fixations, or proportion of regressive saccades when reading typical black text aloud on backgrounds of different luminance. However, there was a small but significant increase in saccade amplitude as background luminance increased and increased fixation durations. Another study compared how various sources of illumination impact the reading ability of adults. Again, there was a lack of significant correlation between illumination and reading speed (Ram and Bhardwaj, 2018). Overall, it seems that, at least in comparison to blur and contrast, luminance changes in the photopic range do not seem to greatly impact reading ability.

These studies demonstrate the adaptivity of human vision to various luminance conditions. While factors such as contrast and blur seem to greatly impact reading ability beyond critical points in normal human vision, luminance does not fall to the same effect. Some studies demonstrate that decreasing the luminance of visual information shown to the fellow eye helps reduce the binocular imbalance and promote binocular fusion in amblyopia (Min et al., 2022; Zhou et al., 2024). Furthermore, one study reports that individuals with strabismic amblyopia had a normal contrast threshold under scotopic and mesopic conditions, but an altered contrast threshold in photopic conditions (Hess and Howell, 1978).

## 6 Reading is impacted by amblyopia

Noting that various image manipulations, such as low contrast and blur, can affect reading and occur naturally in amblyopia, many studies have focused on how this condition itself can impact reading ability. They determined people with amblyopia present slower reading times, due to altered eye movements and crowding in the fovea which impact their overall reading capability (Kanonidou et al., 2010; Kelly et al., 2015; Stifter et al., 2005; Bhutada et al., 2022; Kelly et al., 2017; Buczkowska and Bogdan, 2017; Kugathasan et al., 2019; Levi et al., 2007; Kanonidou et al., 2014).

### 6.1 Reading accuracy and speed

Amblyopes have been found to read more slowly compared to the normal population (Kanonidou et al., 2010; Kelly et al., 2015; Stifter et al., 2005; Bhutada et al., 2022; Kelly et al., 2017; Buczkowska and Bogdan, 2017; Kugathasan et al., 2019). During silent reading, mean reading speeds of individuals with amblyopia were discovered to be significantly slower than controls in binocular conditions. The average binocular reading speed of amblyopic children and adults was 30% slower than controls which read on average at 235.5 wpm (Bhutada et al., 2022; Kelly et al., 2017; Kanonidou et al., 2010; Kelly et al., 2015). This was also the case for monocular reading conditions, where the average reading speeds for controls in each eye across studies were approximately 1.5 times faster than the average amblyopic reading speeds binocularly. The average dominant eye reading speed was 1.4 times faster than the fellow eye and 1.7 times faster than amblyopic eye monocular reading. The average nondominant eye reading speed was also 1.7 times faster than amblyopic eye average monocular reading speed (Kanonidou et al., 2010; Bhutada et al., 2022; Stifter et al., 2005). Furthermore, for amblyopes, average reading speed of the amblyopic eye was found to be slower in comparison to the non-amblyopic eye and binocular reading. Significantly longer fixation durations and higher numbers of regressive saccades made per line were the oculomotor parameters that particularly affected the reading speed (Kanonidou et al., 2010). This suggests that altered eye movements in amblyopia may affect reading speed and overall reading ability. Stifter et al. (2005) also demonstrated that even in children with microstrabismic amblyopia reading aloud, group averages of amblyopic eye maximum reading speeds were significantly slower than those of the fellow eye.

Bhutada et al. (2022) also demonstrated that adult and child amblyopic participants, with various types of amblyopia, had slower binocular and monocular reading speeds, in both amblyopic and fellow eyes, than control participants during silent paragraph reading. This slower reading was correlated with visual acuity and stereoacuity. In particular, it was anisometropic and mixed amblyopes who had significantly reduced reading speeds compared to controls during monocular reading with the amblyopic eye (anisometropic 53% and mixed 58% the speed of controls) as well as during binocular reading (anisometropic 64% and mixed 57% the speed of controls). Whereas the same effect was not shown in strabismic amblyopes (binocular and monocular with amblyopic eye was 72% the speed of controls). Perhaps this discrepancy is related to the higher proportion of severe mixed amblyopes and moderate anisometropic amblyopes, in comparison to the strabismic amblyopic group which was comprised of more mild and mild treated participants. Kelly et al. (2017) reported reduced reading speed in anisometropic amblyopic children. While controls and amblyopic participants did not differ in text comprehension, amblyopic children read slower and made more forward saccades than both controls, and the nonamblyopic anisometropic children. Accordingly, these factors were found to be significantly correlated. Number of regressive saccades and fellow eye fixation instability during binocular viewing also correlated with reading speed even though there were no differences between groups in these two factors. Similar results were discovered in a group of anisometropic amblyopic children reading in Polish. Compared to controls, the amblyopic children read 30 words less on average demonstrating much slower reading overall (Buczkowska and Bogdan, 2017).

Interestingly, children who were treated for either anisometropic or strabismic amblyopia through methods including monocular occlusion, glasses and/or surgery showed no significant differences from controls in aloud binocular paragraph reading rate, accuracy, and comprehension. Though 29% of amblyopic children still demonstrated overall worse binocular oral reading than controls (Kugathasan et al., 2019). Another study comparing amblyopic children treated with patching to controls found that binocular and monocular reading speeds and reading acuity were not significantly different between groups and nor was maximum reading speed (Fernandes and Ferraz, 2022). Even comparing treated and fellow eyes within participants did not yield significantly different reading speeds. However, only reading speed at a print size of 0.7 logMAR was included in the study. Thus, the differences in reading between a treated amblyopic population and controls may not have been fully revealed. This result opposes that of an earlier study by Repka et al. (2008), where amblyopic and fellow eyes, of anisometropic, strabismic or mixed amblyopic children treated via patching or atropine drops, were compared on reading scores from the GORT-4, a normalized test in which print size of paragraph text read monocularly is based on the appropriate size for the age of the child. They discovered that monocular reading speed and accuracy was worse in the treated amblyopic eye than the fellow eye. However, the discrepancies between the results of these studies may be due to Fernandes and Ferraz (2022), including only 10 strabismic amblyopes in their data set, whereas Repka et al. (2008), included 79 amblyopic children of different amblyopia types.

In general, the literature demonstrates that amblyopes read at a slower rate than controls binocularly and monocularly. In monocular reading the amblyopic eye has a slower rate than the fellow eye. Treatment for amblyopia in childhood appeared to improve reading speed in all but one study, demonstrating the importance of, starting treatment for the condition as soon as possible to prevent long-term reading impacts.

### 6.2 Text print size

Literature has described difficulties amblyopes face in reading fine print with their amblyopic eye (Levi et al., 2007). Typically reading speed increases quickly with text size until it reaches a plateau, where larger text sizes have no effect on the reading rate. The point at which a reading rate curve reaches this plateau, or where the text size no longer affects reading speed, is referred to as the critical print size (Pelli et al., 2007). Studies find that amblyopes tend to have higher critical print sizes in the amblyopic eye (Levi et al., 2007). It has also been suggested that regardless of font size, individuals with strabismic amblyopia tend to read slower than controls and have longer fixation periods. On average, adult amblyopes were found to read one line less of text than control participants during silent paragraph reading in different font sizes, both binocularly and monocularly, this effect became more apparent as font size became smaller. Thus, it follows that the duration of fixation periods was also significantly longer for amblyopes than for controls. However, this was only when font sizes were much smaller and only the amblyopic eye alone was reading (Kanonidou et al., 2014). Stifter et al. (2005) discovered that in even in children with microstrabismic amblyopia, both amblyopic and fellow eye reading speed decreased as font sizes became smaller. Though even in average and larger font sizes, amblyopic eye reading was still slower than that of the fellow eye. As such, when comparing the critical print size of the amblyopic and fellow eyes between individuals, the size of text required for optimal reading was much larger in the amblyopic eye. Overall, the literature reveals that the slower reading in amblyopia is amplified as print sizes become smaller, both compared to controls, and within amblyopes themselves between the amblyopic and fellow eye.

## 7 Altered eye movements in amblyopia during reading

Due to the important role of oculomotor movements in the process of reading, a prominent aspect of the literature investigating reading difficulties, including reductions in reading speed and worse accuracy, in the amblyopic population is related to altered eye movement patterns. Notably, these studies have reported alterations in fixation stability, forward and reverse saccade patterns and amplitudes, as well as the number and length of fixation periods.

### 7.1 Forward/reverse saccade alterations

In general, amblyopes have altered eye movement patterns in various tasks. Niechwiej-Szwedo et al. (2010) reported slower and more random initiation of primary saccades in monocular viewing with the amblyopic eye and binocular viewing in a reaching task. They also found greater variation in saccade amplitudes of amblyopic participants, as well as more corrective saccades made by the fellow eye. Shaikh et al. (2016) found, in a fixational target tracking task, that amblyopic children make less fixational saccades, though they have higher amplitudes, an effect which strengthened with more severe amblyopia. Even during visual search and fixation tasks, amblyopes have an altered pattern of saccades. They were found to make more microsaccades and saccades in both fellow and amblyopic eyes (Chen et al., 2018). These alterations apply to proper reading which heavily involves the use of saccades and fixations to efficiently obtain information from text.

Some studies have specifically investigated how these eye movement patterns change in amblyopia during the process of reading. Kanonidou et al. (2010) compared eye movement patterns of adult strabismic amblyopes and control participants while reading silently binocularly and monocularly. Comparing nonamblyopic to control dominant eye viewing and in the binocular viewing condition, amblyopes made significantly more total number of saccades, including both forward and reverse. In the amblyopic to control nondominant eye and binocular viewing comparisons, amblyopes made significantly more reverse saccades per line. No significant differences in the number of forward saccades made per line in any viewing condition were reported and no significant differences in saccade amplitude were observed.

Bhutada et al. (2022) also found altered saccade patterns during passage reading of adult and child participants with amblyopia compared to controls: Amblyopes made more forward saccades than controls in both binocular and monocular fellow eye reading. While there was a similar trend in the amblyopic eye reading condition, this was not significantly different between groups. Thus, suggesting a possible overcompensation of the fellow eye to help intake visual information; and demonstrating that amblyopia does not only affect the amblyopic eye. They also found that amblyopic participants in all binocular and monocular reading conditions made more reverse saccades than controls. Amongst the amblyopic participants, fixational saccade amplitude was discovered to be higher in the amblyopic eye than the fellow eye. Opposing this result, Kelly et al. (2015, 2017) did not find a significant difference in the number of reverse saccades between amblyopic children and controls when reading paragraphs of text binocularly. The difference between the studies by Kelly et al. (2015, 2017), and Bhutada et al. (2022), that may account for this inconsistency, is the age range which was much wider and older in the later study, 7–51 years old, compared to 7–12 years old and 8–12 years old. This may mean that changes to reverse saccade patterns develop more with age. However, similarly to Bhutada et al. (2022) and Kanonidou et al. (2010), these children also had an increased number of forward saccades (Kelly et al., 2015; Kelly et al., 2017).

Clearly, saccade patterns are altered in amblyopia which these studies reported to relate to slower reading speeds in amblyopia (Bhutada et al., 2022; Kanonidou et al., 2010; Kelly et al., 2015; Kelly et al., 2017). However, they also noted that fixation periods have a large impact on reading speed.

### 7.2 Fixation periods

While proper saccade patterns are important for efficient reading, it is during the fixation periods that individuals receive visual input. As such, changes in fixation duration and stability may have an adverse effect on individuals reading ability. In amblyopia, there are various reports on changes to fixation periods in amblyopic vision and their impact on these individuals’ reading abilities. Kanonidou et al. (2014) found that strabismic amblyopes had more fixations than controls when reading monocularly with the amblyopic eye. In accordance with this, Bhutada et al. (2022) demonstrated that amblyopes had higher fixational instability and longer fixation periods while reading passages of text. In terms of total fixation duration, anisometropic amblyopes had significantly longer fixation durations than controls when comparing monocular amblyopic eye to nondominant eye reading (1.7 times longer). This was not seen in strabismic amblyopes, even though when data was combined for all amblyopia subtypes, amblyopes had fixation durations 1.5 times longer than controls reading binocularly and monocularly with each eye (Bhutada et al., 2022). However, another study by Kelly et al. (2015) did not find any differences in reading rate, saccades and fixation duration based on amblyopia sub-type. Kelly et al. (2015) tested subjects who were on average younger than those tested in Bhutada et al. (2022) which may account for this difference. An earlier study by Kanonidou et al. (2010), showed longer fixation periods, compared to controls, when amblyopes read monocularly with the amblyopic eye, and binocularly. On the other hand, Kelly et al. (2017) found that anisometropic amblyopic children reading silent paragraphs of text binocularly did not have any differences in fixation duration compared to anisometropic only and control children. Though they did find that the reduced reading rate observed in the amblyopic group was significantly correlated with fixation instability in the fellow eye but not the amblyopic eye. This was also believed to cause the increase in forward saccades. The difference in results observed in Kelly et al. (2017) compared to Kanonidou et al. (2014), Bhutada et al. (2022) and Kanonidou et al. (2010) is that the latter studies were focused on adults only or adults and children, whereas Kelly et al. (2017) was focused on only anisometropic amblyopic children. Thus, demonstrating that even within amblyopic populations, the characteristics which cause reading challenges may differ. Furthermore, there are other factors besides oculomotor patterns, such as text crowding, which have an impact on the reading ability of adults and children with various types of amblyopia.

## 8 Amblyopia causes crowding of text during reading

Crowding is the phenomenon that occurs when the ability to determine a target that is detectable in isolation is hindered by flanking stimuli (Stuart and Burian, 1962), and it is particularly prominent in peripheral vision (Lettvin, 1976). During reading, crowding interrupts the ability to distinguish individual letters and words, especially when they are closely spaced (Bricolo et al., 2015; He and Legge, 2017; Liu et al., 2017; Martelli et al., 2009; Pelli et al., 2007). Pelli et al. (2007) find that reading speed is limited by the extent of crowding, demonstrating that it is directly proportional to the number of letters that fit in the uncrowded visual span. Bricolo et al. (2015) add to this finding that, with increased text spacing, there is shorter first fixation periods on the text, even though gaze duration can increase. In general, crowded stimuli do not lose features such as contrast, but simply become cluttered together, making it difficult to identify differences between target and flanker, target position and orientation (Levi, 2011).

Crowding is particularly prominent in individuals with amblyopia, as many have reported strong crowding not only in the periphery, but in the amblyopic fovea (Bonneh et al., 2007; Chung et al., 2008; Irvine, 1948; Levi and Klein, 1985; Levi et al., 2007; O'Boyle et al., 2017). Studies have found that amblyopic foveal vision is more like that of control peripheral vision than control central vision, in terms of crowding (Hariharan et al., 2005; Levi et al., 2007). Hariharan et al. (2005) found that in both the control periphery and amblyopic fovea, crowding occurs at larger spatial distances than in the control fovea and is independent of stimulus size. Levi et al. (2007) showed that, in foveal vision, individuals with amblyopia had a more difficult time identifying a letter correctly when they were flanked by other letters compared to when the letter was presented in isolation. They indeed suggest that it is particularly the higher levels of crowding in amblyopia that affects reading ability. A later study by Norgett and Siderov (2017), confirmed this effect finding worse visual acuity in the amblyopic eye when letters were flanked, rather than being presented alone. They add that there was a lot of differences in crowding amongst amblyopic participants, though on average crowding was greater for amblyopes when the target was flanked by letters, rather than bars, and more letters presented linearly made crowding even worse. However, to our knowledge, no study has precisely quantified the relationship between crowding and reading speed in amblyopia.

One suggested mechanism behind crowding in amblyopia is abnormal flanker-target integration following the detection of stimuli features (Hariharan et al., 2005). Other hypotheses for the mechanisms of crowding in amblyopia include bigger cortical receptive fields (Flom et al., 1963) and irregular inhibitory interactions (Bonneh et al., 2004), as discussed by Chung et al. (2008). Though their more recent results align most with the target-flanker interaction theory over a wide spatial range (Chung et al., 2008). Levi et al. (2007) suggest that it is crowding rather than poor visual acuity which impacts reading ability in amblyopia. As once the letters of the text are appropriately spaced, the reading rates of both amblyopic and non-amblyopic eyes become much more similar. Kanonidou et al. (2014) also found that font size has an impact on how close the letters appear together, and smaller size fonts have more crowding. Using eye tracking, they found that when strabismic amblyopic participants read smaller text monocularly with their amblyopic eye, they had longer fixation periods. They suggested this may be due to crowding in the fovea causing an increased processing duration.

In general, the literature demonstrates that the increased crowding in individuals with amblyopia is one of the many features of the condition that may impact reading ability.

## 9 Improving amblyopic vision through reading

In this review, we not only discussed the advantages of binocular reading and how this is impacted in reduced viewing conditions but also highlighted that reading deficits in amblyopia are present both monocularly in each eye and binocularly. Reading speeds are slower in amblyopes even when reading with both eyes (Bhutada et al., 2022; Kelly et al., 2017; Kugathasan et al., 2019). Their eye movements are also altered binocularly (Chen et al., 2018; Kanonidou et al., 2010; Bhutada et al., 2022) and even in the fellow eye (Bhutada et al., 2022). The increased crowding in the amblyopic eye fovea also contributes to a reduced equality between the eyes during reading which interrupts the ability to efficiently process text, an advantage only binocular reading provides (Nikolova et al., 2017; Jainta and Joss, 2019). This suggests that reading in amblyopia is affected by impairments to the visual system as a whole and therefore therapies developed for this condition should address this issue binocularly.

**Figure 2:**
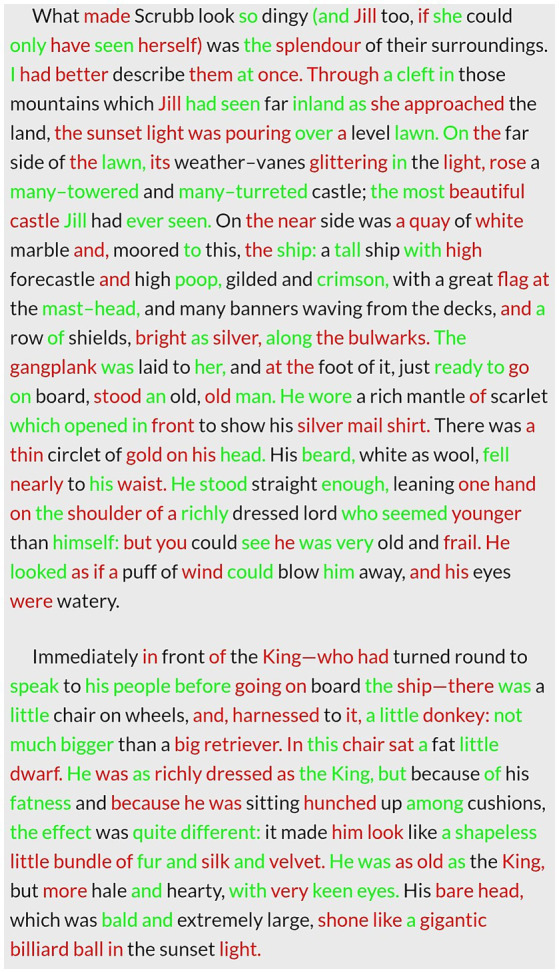
Example of dichoptic e-book reading application (DEBRA). Text is shown in dichoptic anaglyph format. Using red/green anaglyph glasses, some words would only be seen by one eye (red/green) or by both eyes (black text). Text sourced from *The Chronicles of Narnia: The Silver Chair* by C. S Lewis. Figure adapted from Dranitsaris et al. (2026).

New treatments for amblyopia have focused on the use of dichoptic tools, where each eye is independently shown different visual input, and the patient is forced to combine information from both eyes (To et al., 2011; Xiao et al., 2020; Li S. L. et al., 2015). So far, video game (To et al., 2011) and movie (Li S. L. et al., 2015; Xiao et al., 2020) based dichoptic therapies have been introduced. We propose that the dichoptic approach could be applied to a reading-based technology (Dranitsaris et al., 2026), which may not only be able to target binocular fusion but may also be a way to target the altered eye movement patterns in reading. Text would be presented dichoptically to force the reader to use both eyes at the same time to read all the text on screen (Figure 2; Dranitsaris et al., 2026). Although such a tool has yet to be validated, it could help relieve the interocular suppression of the fellow eye on the amblyopic eye as the visual fields are separated, but also uniquely targets reading, for which the effects of dichoptic presentation have yet to be investigated. Indeed, studies have shown that individuals with central vision loss who experience binocular inhibition (25–41%) have significantly slower reading speeds (average of 48.5 wpm slower) compared to those who instead have binocular summation or equality (Tarita-Nistor et al., 2013; Silvestri et al., 2020). Due to the binocular nature of the tool, it is possible that it may also impact the stereovision of individuals with amblyopia. This has been found after training on other dichoptic therapies led to a significant improvement in stereopsis (Wygnanski-Jaffe et al., 2023; Li et al., 2013; Ojiabo and Munsamy, 2022).

As previously discussed, some studies have investigated whether any treatment for amblyopia led to improved reading skills. Kugathasan et al. (2019) found that treated amblyopes between 7 and 17 years old had no significant difference in paragraph reading rate, accuracy and comprehension scores compared to controls. However, there were still deficits that remained in single word reading where controls performed significantly better. It is worth noting that even after treatment, amblyopes were still found to have worse visual acuity in their amblyopic eye. Fernandes and Ferraz (2022) had a similar result of improved reading speed following amblyopia treatment via monocular occlusion. However, to our knowledge, no studies particularly discussed the impact of dichoptic treatments on reading skill improvement.

Binocular and dichoptic protocol studies for amblyopia have however discussed the improvement of vision aspects related to reading ability such as visuomotor (eye movements), fine motor skills (hand-eye coordination) and binocular integration. One study found that after training on a dichoptic video game tool, amblyopic children had improved binocular function and fine motor skills (Webber et al., 2016). Li et al. (2013) also found improved stereoacuity after dichoptic training compared to monocular occlusion therapy in adults, indicating some improved binocularity which is required for stereovision. As such, a dichoptic reading tool might distinctly be able to improve not only monocular visual acuity, but also binocular integration and reading ability, including the visuomotor skills.
